# Psychosocial factors associated with physical activity, weight management, and sleep in adults with hip and knee osteoarthritis: a systematic review

**DOI:** 10.1186/s41927-025-00506-x

**Published:** 2025-05-09

**Authors:** Britt van Dongen, Amber Ronteltap, Bastiaan Cijs, Corelien Kloek, Catherine Bolman, Rik Crutzen

**Affiliations:** 1 Department of Health Promotion, Care and Public Health Research Institute, Maastricht University, Maastricht, The Netherlands; 2 Research Group Innovation of Movement Care, Research Center Healthy and Sustainable Living, HU University of Applied Sciences Utrecht, Utrecht, The Netherlands; 3 Department of Health Psychology, Faculty of Psychology, Open University of the Netherlands, Heerlen, The Netherlands

**Keywords:** Osteoarthritis, Psychosocial determinants, Self-management, Physical activity, Weight management, Sleep

## Abstract

**Background:**

Osteoarthritis (OA) is a chronic disease primarily affecting older adults, mainly impacting the hip and knee joints. The increasing prevalence of OA contributes to rising healthcare demands and costs. Current OA treatment guidelines emphasize the importance of self-management education and guidance, particularly in promoting physical activity and weight management. In addition, improving sleep is crucial for managing OA. Developing effective self-management interventions necessitates a comprehensive understanding of the factors that facilitate these behaviors. Especially for changing health behaviors, it is important to focus on psychosocial factors. Therefore, this systematic review aimed to identify the psychosocial factors associated with physical activity, weight management, and sleep in adults with hip and/or knee OA.

**Methods:**

Five databases (PubMed, Embase, CINAHL, PyschINFO, Web of Science) were searched for observational studies reporting statistics on the association between psychosocial determinants and physical activity, weight management, or sleep in people with OA. The methodological quality was assessed using the Quality Assessment Tool for Observational Studies of the National Heart, Lung, and Blood Institute. After screening 5,812 articles, 31 studies were included for analysis.

**Results:**

The results showed that intention, self-efficacy, and willpower beliefs were positively associated with physical activity. Kinesiophobia, pain catastrophizing and pain-related fear were negatively associated with physical activity. Depressive symptoms, negative affect, pain catastrophizing, and low willpower beliefs were associated with poor weight management. Anxiety, depression, pain anxiety, and post-traumatic stress disorder were related to poor sleep behavior.

**Conclusions:**

This review enhances the understanding of the psychosocial factors underlying physical activity, weight management and sleep in OA. These insights are valuable for developing tailored behavior change interventions aimed at improving physical activity, weight management and sleep in patients with hip and/or knee OA. Future research is warranted to gain more in-depth insights, particularly through longitudinal studies and further research into the psychosocial determinants of sleep, as current literature in this area is limited.

**Supplementary Information:**

The online version contains supplementary material available at 10.1186/s41927-025-00506-x.

## Introduction

Osteoarthritis (OA) is a chronic joint disease affecting mainly older adults [1], mostly impacting the knee joint, followed by the hip [2–4]. In 2020, 595 million people worldwide had OA, marking a 132% increase since 1990 [5]. This rapid rise over the past three decades is due to three main factors: population growth, aging, and obesity [6]. By 2050, the number of cases of knee OA is expected to increase by 74.9% and hip OA by 78.6% [5]. Hip and knee OA cause considerable pain, stiffness, and physical disability [7]. Treatment guidelines for OA recommend starting with less-intense and lower-risk non-surgical treatments before considering costly joint replacements to maintain patients’ quality of life [8].

Primary care for OA typically includes education, medication use, and self-management support [8, 9], which are often applied simultaneously [10]. Self-management interventions have long-term benefits as they address the physical, psychological, and social aspects of living with a chronic condition [11]. Self-management refers to the ability of patients to actively manage their disease, symptoms, and lifestyle changes related to OA [12]. Behavioral interventions that focus on improving self-management primarily target behaviors like physical activity and weight management, as prescribed in OA guidelines [8].

Physical activity, weight management, and sleep are relevant self-management behaviors for patients with OA because of their substantial benefits [10]. Regular physical activity can reduce pain, enhance physical function, and improve quality of life [13]. For individuals with OA and overweight or obesity, weight loss can decrease pain and stiffness, thereby improving physical functioning [14]. Furthermore, weight loss can reduce fear of movement, supporting greater physical ability, independence, and participation in daily and social activities [15]. Although not explicitly included in current guidelines, improving sleep is crucial for OA management due to the high prevalence of poor sleep quality among patients, which is associated with increased inflammation and pain symptoms [16]. Addressing physical activity, weight management, and sleep are therefore essential components of effective OA management.

However, many patients with OA experience difficulty with adherence to physical activity guidelines, proper weight management [17], and adequate sleep [16]. Optimal guidance and self-management improvements can only be achieved by understanding the factors underlying these behaviors [18], which are called determinants of behavior [19]. Within the scope of this article, the term ‘determinants’ refers to psychological constructs that are assumed to (partly) affect behavior and can be changed through behavior change interventions. For example, fear of joint damage, lack of motivation, and depressive symptoms are psychological determinants that negatively impact physical activity, weight management, and healthy sleep behavior [20–22]. Especially for promoting healthy behaviors, it is crucial to target psychological and social determinants due to their direct effect on behavior [23]. In the past decade, there has been growing interest in the correlates of physical activity in adults with rheumatic disorders for the development of interventions [24, 25]. However, to date, a comprehensive understanding of specifically psychosocial determinants of relevant health behaviors in OA patients is missing, due to knowledge that is either lacking or spread across separate studies [26]. Therefore, the aim of the current systematic review is to identify the psychosocial factors associated with physical activity, weight management, and sleep behavior in adults with hip and/or knee osteoarthritis.

## Methods

This study followed the PRISMA (Preferred Reporting Items on Systematic Reviews and Meta-analysis) guidelines. The full protocol for this systematic review had been preregistered within PROSPERO (CRD42024510079). The protocol was amended after adjustments to data analysis methods, whereby data is now analyzed with a best-evidence synthesis rather than being analyzed in separate subgroups of hip and knee OA.

### Eligibility criteria

The eligibility criteria were divided into four search domains: population, determinants, outcome behaviors, and study design.

#### Population

This review focused on an adult study population (≥ 18 years), with hip and/or knee osteoarthritis based on self-reported physician diagnosis, clinical diagnosis by a health professional (e.g., physiotherapist or general practitioner) based on the American College of Radiology criteria [27], or radiographic diagnosis (grade ≥ 2) [28]. Studies among populations with multiple joint-related pathologies (e.g., rheumatoid arthritis, and general OA) were only excluded when participants with hip and/or knee OA were not analyzed separately. Also, studies were excluded in which participants were awaiting or had undergone joint replacement surgery treating OA. This is because people on the waiting list have greater impairments and complaints which may lead to different determinant structures underlying physical activity, weight management, and sleep. Also, people who were awaiting or had recently undergone joint replacement might have received specific therapeutic instructions from their healthcare professionals resulting in different target behaviors.

#### Determinants

Studies were initially included if title and abstract indicated that at least one psychosocial determinant was measured. During the subsequent full-text screening, only studies were included in which at least one psychosocial determinant was analyzed in relationship with one of the outcome behaviors (outlined below).

#### Outcome behaviors

This review focused on three specific outcome behaviors: physical activity, weight management, and sleep. Each outcome behavior comprised various sub-behaviors that constituted the search strategy (Additional file 1). The physical activity behaviors that were included are only behaviors that requires energy expenditure of more than 3 METs [29]. This is based on the WHO guidelines, which are often used in interventions supporting physical activity in patients with OA. These guidelines recommend that adults engage in 150 min of moderate-intensity physical activity and muscle-strengthening activities on minimal two days per week [30]. Examples of sub-behaviors of physical activity are walking measured with daily steps and strength training converted in MET-minutes. Weight management encompasses behaviors aimed at achieving and maintaining a healthy weight. Sub-behaviors of weight management include, for example, energy intake measured by kilocalories and weight control measured by changes in body weight. Sleep behavior consists of the patterns and activities individuals engage in before, during, and after sleep. This includes, for example, sleep hygiene and sleep duration.

Studies were included when outcome data regarding these behaviors was self-reported, observed with an accelerometer or sensor, or physiologically assessed.

#### Study design

This review included observational (cohort and cross-sectional) studies. Experimental studies were also eligible if data was available on participants without exposure to any intervention, such as baseline or control group data. These data had to provide insight into the relationship between psychosocial determinants and outcome behavior for the study to be included. All other study designs were excluded.

#### Additional criteria

Studies reported in languages other than English or Dutch or focused on animals were excluded. There were no restrictions regarding the publication date.

### Information sources

Five databases were searched to ensure comprehensive coverage of all relevant studies: PubMed, Embase, CINAHL, PsycINFO, and Web of Science.

### Search strategy

The search strategies for this systematic review were collaboratively developed with a library information specialist from Maastricht University. The complete search strings are included in Additional file 1. The final search was conducted in February 2024.

### Study selection

The search results were imported into EndNote to eliminate duplicates, and then transferred to Rayyan [31]. Two researchers (BD, BC) independently screened the title and abstract of all search results. The researchers (BD, BC) started with parallel screening sessions of 50 studies until sufficient inter-rater reliability was reached (Kappa > 0.80). However, of these screening sessions, only 5–10% of the studies met the inclusion criteria. In situations with such low inclusion percentages, the Kappa is known to be an unreliable value of inter-rater agreement [32]. Therefore, after the tenth session (Kappa = 0.649), in which different judgments only pointed to ‘misread’, both researchers agreed that they sufficiently aligned on the inclusion or exclusion of the studies. Misreading refers to misinterpreting or reading inaccurately, unintentionally resulting in a study being incorrectly in- or excluded [33]. After sufficient inter-rater agreement, the two researchers were considered interchangeable and continued screening separately. In case of doubts or conflicting judgments, a meeting was scheduled for the researchers to discuss and agree on these studies.

Before screening the full-texts, the researchers (BD, BC) again discussed the eligibility criteria. The additional criterion was that the relationship between at least one psychosocial determinant and one of the outcome behaviors was analyzed. These insights were crucial for achieving the study’s aim. Based on the adapted criteria, two researchers (BD, BC) alternately screened the included studies on a full-text basis. Studies that were difficult to decide on based on the eligibility criteria were discussed afterward until agreement was reached.

### Methodological quality

The methodological quality of the included studies was assessed using the Quality Assessment Tool for Observational Studies from the National Heart, Lung, and Blood Institute (NHLBI) [34]. The NHLBI was developed specifically for evaluating the internal validity and overall quality of observational research studies, making it an ideal tool for this review. The studies were rated based on fourteen criteria. This assessment was performed independently for all included studies by two researchers (BD, BC). After all included studies were reviewed, the final ratings were compared. We focused on four specific criteria, namely whether the exposure of interest was measured before the outcome was measured (item 6), whether the timeframe was sufficient (item 7), and whether the independent and dependent variables were clearly defined, valid, reliable, and implemented consistently (item 9 and 11). These criteria considerably impact both internal and construct validity, which were deemed crucial aspects given this study’s objective. The quality was rated “poor”, “moderate”, or “good”. In case of disagreement, the researchers (BD, BC) reached a consensus for all studies by discussion.

### Data extraction

Data extraction was conducted in Microsoft Excel. Multiple characteristics were extracted from all included studies, namely first author, year of publication, country of study, study design, sample size, and details about the study population. Then, data from each analyzed determinant was extracted separately for physical activity, weight management, and sleep: psychosocial determinant, measurement of determinant, measurement of outcome behavior, effect size of analysis (e.g., correlation coefficient, odds ratio), and strength of association. Data extraction was done by the first researcher (BD) and checked by another researcher (BC).

### Data analysis

The results from the data extraction were descriptively and narratively analyzed, supported by tables. Due to the studies’ heterogeneity concerning study population, determinants, and outcome measures, conducting a statistical analysis with the available data was not feasible, and power would be low [35]. As an alternative, a best-evidence synthesis was undertaken to decide the weight of the found evidence systematically [36]. The level of evidence was based on the number of studies, the methodologic quality, and the consistency, which resulted in five levels: strong, moderate, weak, inconclusive, and inconsistent (Table 1) [37, 38].

**Table 1:** Levels of evidence used in the best-evidence synthesis

Level	Evidence
Strong	Generally consistent (non)significant associations found in at least two good-quality cohort studies
Moderate	Generally consistent (non)significant associations in one good-quality cohort study and at least one fair-quality cohort study
Weak	(Non)significant association found in one good-quality or fair-quality study or generally consistent (non)significant associations found in at least two poor-quality studies
Inconclusive	(Non)significant association found in less than two poor-quality studies
Inconsistent	Inconsistent significant findings irrespective of study quality (i.e. <66% of the studies reported consistent findings)

## Results

**Fig. 1:**
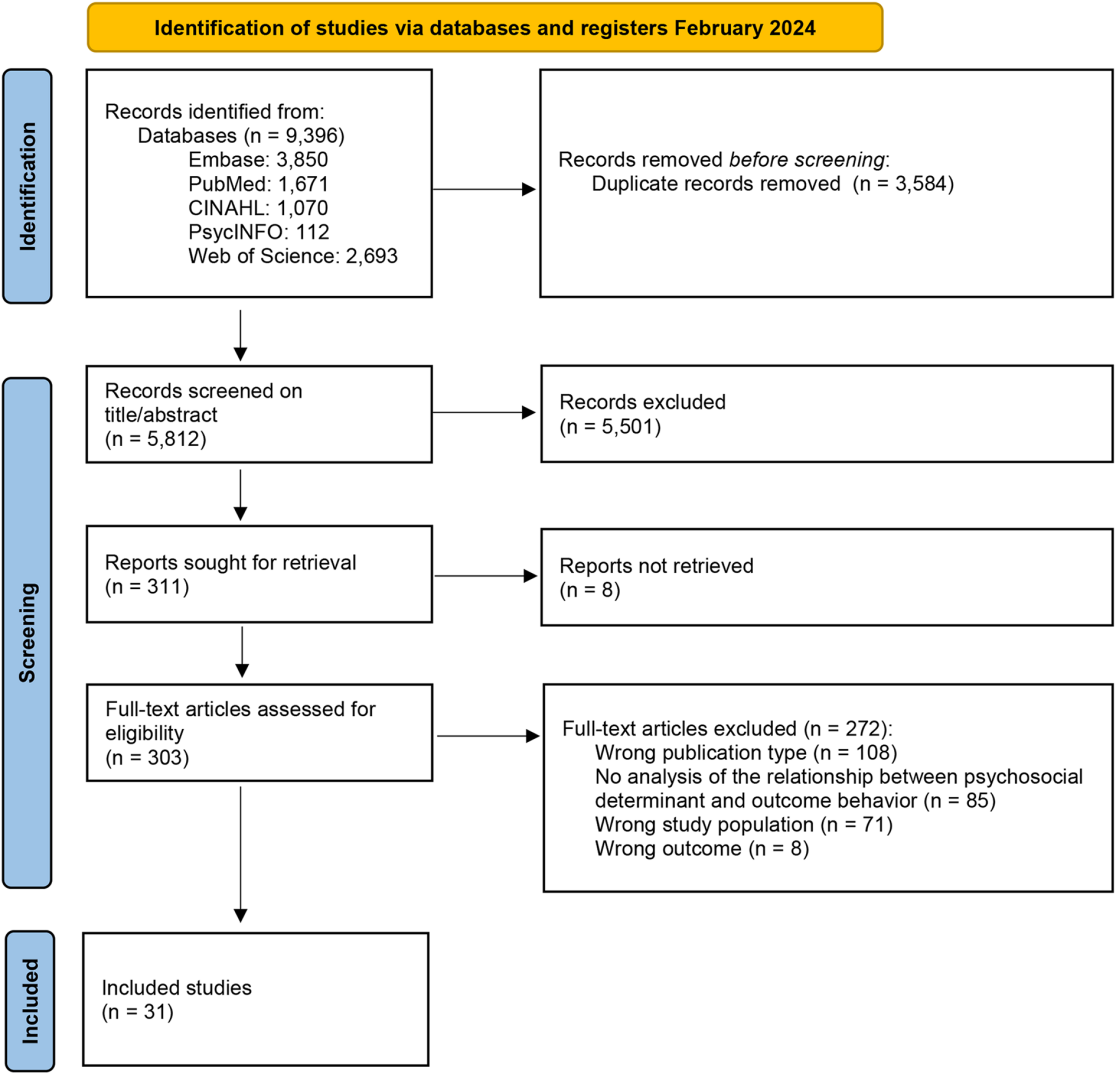
PRISMA Flow Diagram

The search strategy retrieved 9,396 search results from the included databases. In total, 3,584 duplicates were removed, resulting in 5,812 unique references for title and abstract screening. Of those, 303 articles were retrieved for full-text screening, of which 272 were eventually excluded. The main reason for exclusion was that studies were published as conference abstracts only. Other reasons for exclusion are given in the PRISMA flow diagram (Fig. 1). Finally, 31 studies were included with 35 different study populations.

### Study characteristics

The 31 included studies were conducted from 2007 to 2024 in 17 countries (Table 2). Most studies were from North America (*N* = 14) [39–52] and Europe (*N* = 7) [53–59]. Five studies were from Africa [60–64], four from Asia [65–68], and one from Australia [69]. Most studies had a cross-sectional design (*N* = 18) [39, 40, 46, 47, 53, 56, 57, 59–69]. Other designs were longitudinal (*N* = 9) [41–44, 49–52, 54], experimental (*N* = 3) [48, 55, 58], and one study had both cross-sectional and longitudinal designs [45]. The study sample sizes ranged from four to 2,914 participants. Most studies included participants with knee OA only (*N* = 23) [39–42, 44, 45, 47–51, 53, 55, 57, 60–68], seven studies with both knee and hip OA [43, 46, 52, 54, 56, 58, 69], and only one study distinguished participants with either knee or hip OA [59]. Most studies investigated behaviors related to physical activity (*N* = 20) [39, 40, 43, 44, 47, 49–51, 53–59, 65–69], seven investigated behaviors related to weight management [39, 41, 43, 48, 55, 63, 64], and nine investigated behaviors related to sleep [42, 43, 45, 46, 52, 60–63].

**Table 2:** Study characteristics of included studies (*N* = 31)

First author (publication year)	Country^1^	Study design	Study outcome^2^	*N*	Gender (% men)	Age (M ± SD)	Education (% below college degree)	Knee and/or hip OA
Acar (2022) [53]	Germany	Cross-sectional	PA	60	NR	64 ± 11	NR	Knee
Akintayo (2019) [60]	Nigeria	Cross-sectional	S	205	16.4	59.9 ± 10.6	NR	Knee
Aydemir (2022) [39]	USA	Cross-sectional	PA, WM	37	32.4	58.8 ± 8.6	NR	Knee
Aydemir (2023) [40]	USA	Cross-sectional	PA	40	40.0	57.0 ± 8.9	NR	Knee
Choi (2014) [41]	USA	Longitudinal	WM	54	16.4	62.7 ± 9.3	26.0	Knee
Degerstedt (2020) [54]	Sweden	Longitudinal	PA	2,914	28.3	64.7 ± NR	71.8	Both
Di Maio (2020) [55]	Germany	Experimental	PA, WM	243	37.4	65.5 ± 0.5	52.7	Knee
Duarte (2022) [56]	Portugal	Cross-sectional	PA	41	26.8	66.9 ± 8.1	68.3	Both
Fawzy (2022) [61]	Egypt	Cross-sectional	S	20	20.0	43.2 ± 6.5	NR	Knee
Goff (2024) [65]	Singapore	Cross-sectional	PA	425	31.6	63.6 ± 8.0	88.0	Knee
Hamdi (2021) [62]	Tunis	Cross-sectional	S	40	42.0	57.5 ± 12.9	NR	Knee
Hanrungcharatorn (2017) [66]	Thailand	Cross-sectional	PA	242	None	65.1 ± NR	83.5	Knee
Hawker (2010) [52]	Canada	Longitudinal	S	577	22.3	77.8 ± 7.0	NR	Both
Heesch (2011) [69]	Australia	Cross-sectional	PA	485	39.6	68.0 ± 10.6	82.1	Both
Hsu (2022) [67]	Taiwan	Cross-sectional	PA	188	29.8	69.4 ± 7.9	NR	Knee
Kilinc (2019) [57]	Turkey	Cross-sectional	PA	200	40.0	53.2 ± 6.0	NR	Knee
Mahgoub (2020a) [63]	Egypt	Cross-sectional	WM, S	59	None	53.4 ± 7.2	NR	Knee
Mahgoub (2020b) [63]	Egypt	Cross-sectional	WM, S	32	None	37.5 ± 6.9	NR	Knee
Martire (2013) [42]	USA	Longitudinal	S	138	42.0	65.4 ± 9.5	NR	Knee
Murphy (2013) [43]	USA	Longitudinal	PA, WM, S	172	37.8	72.0 ± 6.0	NR	Both
Nemati (2023a) [44]	USA	Longitudinal	PA	2,088	44.9	68.6 ± 9.0	36.7	Knee
Nemati (2023b) [44]	USA	Longitudinal	PA	515	28.9	65.9 ± 8.4	59.1	Knee
O’Brien (2016) [58]	UK	Experimental	PA	4	50.0	58.5 ± NR	NR	Both
Odole (2022) [64]	Nigeria	Cross-sectional	WM	77	19.5	58.0 ± 12.5	NR	Knee
Parmelee (2015a) [45]	USA	Cross-sectional	S	367	36.2	67.0 ± 9.7	49.6	Knee
Parmelee (2015b) [45]	USA	Longitudinal	S	288	34.0	68.3 ± 9.5	45.5	Knee
Rosemann (2007a) [59]	Germany	Cross-sectional	PA	427	44.7	64.8 ± NR	NR	Hip
Rosemann (2007b) [59]	Germany	Cross-sectional	PA	594	26.3	66.8 ± NR	NR	Knee
Taylor (2018) [46]	USA	Cross-sectional	S	300	90.7	61.1 ± 9.2	27.0	Both
Uritani (2020) [68]	Japan	Cross-sectional	PA	167	37.0	62.2 ± 7.5	48.0	Knee
White (2012) [47]	USA	Cross-sectional	PA	1,018	40.0	63.1 ± 7.8	57.0	Knee
Wolf (2010) [48]	USA	Experimental	WM	111	87.0	68.0 ± 8.4	NR	Knee
Zhaoyang (2019) [49]	USA	Longitudinal	PA	143	42.0	65.4 ± 9.5	NR	Knee
Zhaoyang (2020) [50]	USA	Longitudinal	PA	143	42.0	65.4 ± 9.5	NR	Knee
Zhaoyang (2017) [51]	USA	Longitudinal	PA	135	43.7	65.7 ± 9.8	NR	Knee

NR = Not reported

^1^Country: USA = United States of America, UK = United Kingdom

^2^Study outcome: PA = physical activity, WM = weight management, S = sleep

^3^Mahgoub studied two population segments separately. Mahgoub (2022a) is about participants without fibromyalgia and Mahgoub (2022b) is about participants with fibromyalgia

^4^Nemati studied two population segments separately. Nemati (2023a) is about White participants and Nemati (2023b) is about Black participants

### Methodological quality assessment

All studies were critically evaluated using the fourteen criteria of the NHLBI tool, with a particular focus on criteria 6, 7, 9, and 11 (Additional file 2). Most studies were cross-sectional and only had measurements at the same time point. Eleven studies were longitudinal and measured the exposure of interest before the outcome was measured [41, 43, 45, 48–51, 54, 55, 58, 68]. Of these longitudinal studies, only four had a sufficient timeframe (varying from six weeks to one year) so that one could reasonably expect to see an association between exposure and outcome if it existed [45, 48, 54, 58]. In six of the 31 studies, the independent variables were not clearly defined, valid, or reliable [41, 48, 49, 51, 57, 58]. In three studies, the dependent variables were not clearly defined, valid, or reliable [42, 52, 54].

Based on the methodological quality assessment, seven of the 31 studies (22.6%) scored poorly [42, 47, 51, 52, 57, 58, 61]. Since this review aims to provide a comprehensive overview of all existing studies within the scope of the review, excluding these studies was not considered. However, due to the risk of bias in these studies, their results were interpreted with caution. Excluding these studies would not have substantially altered the overall findings.

### Physical activity

Twenty studies analyzed an association between one or more psychosocial determinants and physical activity (PA) behavior (Additional file 3). In total, thirteen psychosocial determinants were analyzed: barriers to physical activity, benefits of physical activity, depressive symptoms, intention, kinesiophobia, negative affect, pain catastrophizing, pain-related fear, perceived controllability, positive affect, self-efficacy, social support, and willpower beliefs. PA behavior consisted of meeting guideline recommendations, activity counts per minute, daily MVPA, daily step count, PASE scores, MET minutes per week, or UCLA activity scores. There were 57 different analyses, of which 30 showed significant associations with or effects on PA. Of these associations, PA behavior was significantly associated with intention, kinesiophobia, pain catastrophizing, pain-related fear, self-efficacy, and willpower beliefs (Table 3).

Eight studies investigated the relationship between depressive symptoms and PA [43, 44, 47, 55, 57, 59, 67, 68]. Out of eleven associations, three had a negative association [44, 47, 57] and two had a positive association [59] ranging in strength from weak to moderate. Nemati, Keith and Kaushal [44] also showed this relationship over time in a study with fair methodological quality. Six studies analyzed self-efficacy [51, 54, 58, 65, 68, 69], of which two poor quality studies and two fair quality studies showed weak positive associations [51, 54, 58, 65]. Kinesiophobia was analyzed by six studies [39, 40, 53, 57, 65, 68], of which four fair quality studies and one poor quality study showed weak or strong negative associations [39, 40, 57, 65, 68]. In one fair quality study, barriers to and benefits of physical activity showed weak associations with respectively strengthening and stretching exercises [69]. Two studies showed a significant strong relationship: one fair study between intention to exercise and PA and one poor study between intention to walk and daily steps [56, 58]. In a fair and a good quality study, a weak negative relationship was found between pain catastrophizing and PA [50, 68]. Pain-related fear was weakly negatively associated with low and moderate PA according to a fair quality study [66]. One study showed a moderate-to-strong negative relationship between perceived controllability and step count [58]. However, due to the study’s poor quality, this relationship turned out positive two days later raising doubt on the reliability of the results. In a fair quality study, willpower beliefs were positively related to moderate to vigorous PA and daily steps [55]. Negative affect, positive affect, and social support showed no significant association with PA based on two poor, two fair, and one good quality study [47, 49–51, 66].

**Table 3:** Results of the best-evidence synthesis on studies about determinants of physical activity

Determinant	Number of studies (*N*)	Methodological quality			Level of evidence
		Poor	Fair	Good	
Barriers to physical activity	1	-	1	-	Weak
Benefits of physical activity	1	-	1	-	Weak
Depressive symptoms	8	2	5	1	Inconsistent
Intention	2	1	1	-	Weak*
Kinesiophobia	6	1	5	-	Weak*
Negative affect	3	1	1	1	Moderate
Pain catastrophizing	3	-	2	1	Moderate*
Pain-related fear	1	-	1	-	Weak*
Perceived controllability	1	1	-	-	Inconclusive
Positive affect	3	2	1	-	Weak
Self-efficacy	6	2	4	-	Weak*
Social support	2	1	1	-	Weak
Willpower beliefs	1	-	1	-	Weak*

*Generally consistent significant findings (*p* <.05)

### Weight management

The relationship between psychosocial determinants and weight management was analyzed in seven different studies with twenty bivariate and multivariate analyses, of which ten analyses resulted in significant associations (Additional file 3). The psychosocial determinants that were studied included depressive symptoms, kinesiophobia, motivation, negative mood, pain anxiety, pain catastrophizing, self-efficacy, and willpower beliefs. Weight management was measured with BMI, weight loss, calorie, fat, or sugar intake. Depressive symptoms, negative mood, pain catastrophizing, and willpower beliefs were significantly associated with weight management (Table 4).

Four studies investigated the relationship between depressive symptoms and weight management [43, 48, 55, 63]. In three fair quality studies, depressive symptoms were weak to moderately positive related to BMI [55, 63] and moderately negative related to weight loss after 16 or 32 weeks [48]. In one fair quality study, negative mood was associated with lower calorie, fat, and sugar intake [41]. One fair study investigated the associations between BMI and pain catastrophizing and self-efficacy, which were respectively moderately positive and moderately negative associations [64]. Self-efficacy showed no significant association with weight loss after 16 or 32 weeks [48]. Willpower beliefs were negatively associated with BMI [55]. Four fair quality studies showed no relationship between kinesiophobia, motivation, and pain anxiety [39, 48, 63, 64].

**Table 4:** Results of the best-evidence synthesis on studies about determinants of weight management

Determinant	Number of studies (*N*)	Methodological quality			Level of evidence
		Poor	Fair	Good	
Depressive symptoms	4	-	3	1	Moderate*
Kinesiophobia	2	-	2	-	Weak
Motivation	1	-	1	-	Weak
Negative mood	1	-	1	-	Weak*
Pain anxiety	1	-	1	-	Weak
Pain catastrophizing	1	-	1	-	Weak*
Self-efficacy	2	-	2	-	Inconsistent
Willpower beliefs	1	-	1	-	Weak*

*Generally consistent significant findings (*p* <.05)

### Sleep

Nine studies analyzed the relationship between psychosocial determinants and sleep in people with hip or knee OA (Additional file 3). There were fifteen analyses considering five psychosocial determinants: anxiety, couple closeness, depressive symptoms, pain anxiety, and post-traumatic stress disorder. Sleep is measured by sleep problems, sleep quality, sleep efficiency, sleep disturbance, and insomnia. The analyses showed that sleep is significantly associated with anxiety, depressive symptoms, pain anxiety and post-traumatic stress disorder (Table 5).

The relationship between depressive symptoms and sleep was analyzed in eight studies [43, 45, 46, 52, 60–63]. In one poor and two fair quality studies, depressive symptoms were positively associated with poor sleep quality, ranging from weak-to-moderate associations [52, 60, 63]. In one poor and one fair quality study, insomnia and depressive symptoms were positively related, of which one was a strong association [46, 61]. Depressive symptoms were moderately positively associated with sleep problems [62] and weakly related to sleep disturbance [45]. Parmelee, Tighe and Dautovich [45] also showed that an increase in depressive symptoms led to an increase in sleep disturbance after one year. Sleep efficiency was weakly negatively influenced by depressive symptoms [43]. Couple closeness was weakly positively related to sleep quality [42]. One good quality study analyzed the relationship between anxiety and sleep problems, which was a strong positive relation [62]. In one fair quality study, pain anxiety had a moderate positive association with poor sleep quality [63]. Post-traumatic stress disorder was analyzed in one fair quality study and was positively associated with insomnia [46].

**Table 5:** Results of the best-evidence synthesis on studies about determinants of sleep

Determinant	Number of studies (*N*)	Methodological quality			Level of evidence
		Poor	Fair	Good	
Anxiety	1	-	1	-	Weak*
Couple closeness	1	1	-	-	Inconclusive
Depressive symptoms	8	2	4	2	Strong*
Pain anxiety	1	-	1	-	Weak*
Post-traumatic stress disorder	1	-	1	-	Weak*

*Generally consistent significant findings (*p* <.05)

## Discussion

This study investigated the psychosocial determinants associated with physical activity, weight management, and sleep in adults with hip and/or knee OA. The results showed that kinesiophobia was negatively related to physical activity – the more fear of movement people with OA feel, the less physically active they are. In addition, intention was positively associated with physical activity– the higher the intention to be physically active, the more physically active people with OA are. Furthermore, self-efficacy and willpower beliefs were positively associated with physical activity, and pain catastrophizing and pain-related fear were negatively related to physical activity. Modest evidence was found for the associations between weight management and depressive symptoms, negative mood, pain catastrophizing, and willpower beliefs. Finally, there was sufficient evidence supporting the association between sleep and anxiety, depressive symptoms, pain anxiety, and post-traumatic stress disorder.

Overall, the findings align with results from studies with populations similar to patients with OA. In patients with one or more cardiovascular risk factors, intention and self-efficacy positively affect physical activity [70]. Also, a recent systematic review examining physical activity determinants in individuals with cardiovascular disease highlighted the substantial negative impact of fear of engaging in physical activity on exercise behavior [71], which aligns with our findings. Likewise, previous studies showed that the level of physical activity of older adults with chronic pain was found to be negatively associated with fear-avoidance beliefs [72, 73]. As explained in the Fear-Avoidance model, the response to pain after physical activities can be misinterpreted as a catastrophe, which leads to an excessive fear of physical movements such that people avoid those physical activities [74]. Moreover, this decrease in physical activity that is related to avoidance may lead to physical deconditioning that in turn may further exacerbate pain and fear of movement. Therefore, these fear-avoidance beliefs need to be addressed in behavioral interventions to increase physical activity in patients with OA.

Previous research on psychosocial determinants associated with weight management and sleep within the OA population is limited. Considering weight management, most studies on psychosocial determinants focus on overweight or obese populations without OA. The results of those studies are only partly in line with our results. For example, a systematic review has provided strong evidence for self-efficacy as a positive predictor of weight loss maintenance in overweight and obese individuals [75]. In addition, obese rheumatoid arthritis patients who have low levels of self-efficacy and high levels of pain catastrophizing were more likely to engage in overeating [76]. However, current results on the relationship between self-efficacy and weight management were inconsistent. Although psychosocial determinants related to sleep have also not been extensively studied in the OA population solely, the findings align with similar studies in older adults with chronic pain. For instance, this review strengthens the existing evidence for the association between depressive symptoms and sleep complaints in chronic pain patients [77]. Additionally, pain-related anxiety has been found as a predictor of sleep quality in chronic pain patients [78].

Well-known psychological theories emphasize the primary role of intention and self-efficacy in understanding human behavior, such as the Reasoned Action Approach [79] and Social Cognitive Theory [80]. Consequently, given the established relevance of intention and self-efficacy to behavior, it is unsurprising that these factors are also associated with behaviors in adults with hip and knee OA. Besides intention and self-efficacy, other key determinants influencing behavior are outcome expectations and behavioral capabilities, among which knowledge and skills [81, 82]. However, in this review, only self-efficacy was identified as a determinant of physical activity. This discrepancy may be attributed to the uncommon practice of assessing certain psychological constructs quantitatively in this medical domain. Interestingly, a systematic review focusing on qualitative evidence related to barriers and facilitators of physical activity in knee and hip OA did uncover these psychological constructs [83]. Also, qualitative studies with older adults with knee OA identified further psychosocial factors related to weight control, such as knowledge, skills, and self-perception [22, 84]. Since our review excluded qualitative studies, it is plausible that these additional determinants have not been uncovered.

As always, this systematic review has its limitations. One limitation is the continued screening of studies by a single researcher, which allowed for potential misreading. This could result in the incorrect assessment of studies. However, considering the numerous prior sessions that refined the eligibility criteria and the consensus achieved, it is unlikely that any misreading affected the study selection. Furthermore, we did not hand-search the reference lists of the included studies for any potentially eligible studies. This could have made the search more comprehensive and can be considered a limitation. Another methodological limitation concerns the criteria used for the study’s quality. The NHLBI tool lacked objective guidelines for assessing methodological quality and subsequently the levels of evidence for the best evidence synthesis. Consequently, the levels of evidence were based on previous reviews on OA-related factors [37, 38]. Despite differences in the criteria used for methodologic quality, the weight was also on internal validity and the informativeness of the study [85], thereby validating the current conclusions regarding the level of evidence. Another potential limitation concerns the eligibility criteria, which included only participants with confirmed knee or hip OA diagnoses. It is possible that some determinants could also have emerged in patients with hip and knee OA complaints, not formally diagnosed by a healthcare professional. While this specificity ensures reliable results for this specific population, it may be that certain psychological factors have not been extensively investigated within this population separately. This may have led to a relatively limited number of psychosocial determinants. As previously mentioned, we may also have missed relevant psychosocial determinants by excluding qualitative studies as psychosocial determinants are usually not standard outcome measures in most OA studies. However, this also strengthens this review by providing empirical evidence that facilitates comparison of the associations found.

The findings in this review can be used to develop and tailor interventions to improve physical activity, weight management, and sleep behavior in patients with hip and/or knee OA. For instance, promoting self-efficacy or diminishing fear-avoidance beliefs can enhance adherence to physical activity guidelines. In addition, insights into relevant determinants associated with these behaviors can help healthcare professionals understand the behavior of patients with OA. However, given the predominantly weak evidence regarding the relationships between psychosocial determinants and health behaviors in the OA population, the conclusions should be interpreted cautiously.

Future studies can extend current findings with longitudinal research as the majority of the included studies had a cross-sectional design. Given that this study design does not provide evidence for the direction of the relationship [86], causality remains unaddressed and warrants the use of other tools, such as directed acyclic graphs [87]. By focusing on longitudinal research, future studies can provide a deeper understanding of the causal relationships between psychosocial factors and physical activity, weight management, and sleep behaviors. Given that these behaviors are often targeted simultaneously in interventions [88, 89], research on the interaction between psychosocial factors and behaviors is also warranted. Additionally, future research should focus on further quantitatively exploring the relationship between psychosocial determinants and weight management and sleep, as there is limited research in this area.

## Conclusions

This review systematically reports existing evidence on the psychosocial factors associated with physical activity, weight management, and sleep in adults with hip and/or knee OA. As the included studies differed in quality, conclusions about the strength or direction of the relationships should be drawn cautiously. Psychosocial determinants for developing tailored behavior change interventions aimed at physical activity, weight management, and sleep are: anxiety, depressive symptoms, intention, kinesiophobia, pain anxiety, pain catastrophizing, self-efficacy, and willpower beliefs. Targeting these psychosocial determinants is a promising strategy for enhancing behavior change and self-management of adults with hip and/or knee OA, thereby improving their quality of life.

## Electronic supplementary material

Below is the link to the electronic supplementary material.

Supplementary Material 1: Additional file 1 - Search strings.pdf. Complete search strategy used per database

Supplementary Material 2: Additional file 2 - Methodological quality.pdf. Assessment of methodological quality of included studies using the NHLBI tool by researcher BD

Supplementary Material 3: Additional file 3 - Psychosocial determinants.pdf. Extracted data of study results concerning the relationship between a psychosocial determinant and one of the outcome behaviors (physical activity, weight management or sleep)

## Data Availability

The datasets used and/or analyzed during the current study are available from the corresponding author on reasonable request.

## Abbreviations

OA: Osteoarthritis
PRISMA: Preferred Reporting Items on Systematic Reviews and Meta-analysis
MET: Metabolic Equivalent
NHLBI: National Heart, Lung and Blood Institute
PA: Physical Activity
MVPA: Moderate to Vigorous Physical Activity
PASE: Physical Activity Scale for the Elderly
UCLA: University of California, Los Angeles
BMI: Body Mass Index
