# Supplementary material for: Psychosocial factors associated with physical activity, weight management, and sleep in adults with hip and knee osteoarthritis: a systematic review
Source: BMC Rheumatol. 2025 May 9;9:51. doi: 10.1186/s41927-025-00506-x (PMC12063410; doi:10.1186/s41927-025-00506-x)
Supplement: Supplementary file 1 — Supplementary Material 1: Additional file 1 - Search strings.pdf. Complete search strategy used per database [file 41927_2025_506_MOESM1_ESM.pdf]

Additional file 1. Search strings and search results

PubMed 06/02/2024		
	**Search query**	**Search results**
**Domain #1**	"Osteoarthritis, Hip"[Mesh] OR "Osteoarthritis, Knee"[Mesh] OR (("Hip Joint"[Mesh:NoExp] OR "Hip"[Mesh] OR "Knee Joint"[Mesh:NoExp] OR "Knee"[Mesh]) AND "Osteoarthritis"[Mesh:NoExp]) OR ((Hip[tiab] OR hips[tiab] OR Knee[tiab] OR knees[tiab] OR "lower limb*"[tiab] OR "lower extremit*"[tiab]) AND (Osteoarthr*[tiab] OR "Osteo-arthr*"[tiab] OR Arthrosis[tiab] OR Arthroses[tiab] OR "Degenerative arthr*"[tiab] OR Coxarthr*[tiab] OR "Cox-arthr*"[tiab] OR Gonarthr*[tiab]))	68,147
**Determinant #2**	"Risk Factors"[Mesh:NoExp] OR Risk*[tiab] OR Factor*[tiab] OR Facilitat*[tiab] OR Barrier*[tiab] OR Determin*[tiab] OR Predict*[tiab]	11,197,764
**Outcome Weight Management #3**	"Diet"[MeSH:NoExp] OR "Diet, Healthy"[MeSH] OR "Diet, Reducing"[MeSH] OR "Energy intake"[MeSH] OR "Eating habit*"[tiab] OR "Eating behavi*"[tiab] OR "Weight management"[tiab:~2] OR "Weight maintenance"[tiab:~2] OR "Weight reduction"[tiab:~2] OR "Weight control"[tiab:~2] OR "Weight loss"[tiab:~2] OR "Lost weight"[tiab] OR ("Weight"[tiab] AND "Adherence"[tiab])	397,816
**Outcome Physical Activity #4**	"Exercise"[MeSH:NoExp] OR "Resistance training"[MeSH] OR "Physical activit*"[tiab] OR "Physical activity adherence"[tiab:~4] OR "Motor activit*"[tiab] OR "Physical exercise"[tiab] OR "Home-based exercise"[tiab] OR "Isometric exercise"[tiab] OR "Aerobic exercise"[tiab] OR "Exercise training"[tiab] OR "Exercise program*"[tiab] OR "Exercise adherence"[tiab:~4] OR "Strength program*"[tiab] OR "Resistance training*"[tiab] OR "Muscle training"[tiab] OR "Strength training"[tiab]	317,751
**Outcome Sleep Quality #5**	"Sleep Hygiene"[MeSH:NoExp] OR Sleep[MeSH:NoExp] OR "Sleep Deprivation"[MeSH] OR "Sleep quality"[MeSH] OR "Sleep experience*"[tiab] OR "Sleep maintenance"[tiab] OR "Sleep quantity"[tiab] OR "Sleeping behavi*"[tiab] OR "Sleep disturbanc*"[tiab] OR "Sleep habit*" [tiab] OR "Sleep insufficienc*"[tiab] OR "Sleep fragmentation"[tiab] OR "Sleep trouble"[tiab:~2] OR "Sleeping troubles"[tiab:~2] OR "Sleep hygiene"[tiab] OR "Sleep pattern"[tiab:~2] OR "Sleep patterns"[tiab:~2] OR "Sleep deprivation*"[tiab] OR "Sleep quality"[tiab:~2]	118,738
**Other #6**	(Animals[MeSH Terms]) NOT (Humans[MeSH Terms])	5,191,450

**#7**	Review[tiab] OR "Review"[Publication Type] OR "Meta-Analysis as Topic"[MeSH Terms] OR Meta-analysis[tiab] OR "Meta-analysis"[Publication Type] OR "Letter"[Publication Type] OR "Editorial"[Publication Type] OR "Comment"[Publication Type]	6,347,889
**Total**	(#1 AND #2 AND (#3 OR #4 OR #5)) NOT #6<br>((#1 AND #2 AND (#3 OR #4 OR #5)) NOT #6) NOT #7	2,139<br>1,671

**EMBASE 06/02/2024**		
	**Search query**	**Search results**
**Domain<br>#1**	hip osteoarthritis/ OR knee osteoarthritis/ OR ((hip/ OR knee/) AND osteoarthritis/) OR ((hip OR hips OR knee OR knees OR "lower limb*" or "lower extremit*").ti,ab,kw. AND (osteoarthr* or osteo-arthr* OR arthrosis or arthroses OR "degenerative arthr*" OR coxarthr* OR cox-arthr* OR gonarthr*).ti,ab,kw.)	95,202
**Determinant<br>#2**	risk factor/ OR risk*.ti,ab,kw. OR factor*.ti,ab,kw. OR facilitat*.ti,ab,kw. OR barrier*.ti,ab,kw. OR determin*.ti,ab,kw. OR predict*.ti,ab,kw.	14,399,504
**Outcome<br>Weight<br>Management<br>#3**	diet/ OR healthy diet/ OR low calorie diet/ OR caloric intake/ OR "eating habit*".ti,ab,kw. OR "eating behavi*".ti,ab,kw. OR (weight adj2 management).ti,ab,kw. OR (weight adj2 maintenance).ti,ab,kw. OR (weight adj2 reduction).ti,ab,kw. OR (weight adj2 control).ti,ab,kw. OR (weight adj2 loss).ti,ab,kw. OR "lost weight".ti,ab,kw. OR (weight AND adherence).ti,ab,kw.	559,413
**Outcome<br>Physical<br>Activity<br>#4**	exercise/ OR resistance training/ OR "physical activity".ti,ab,kw. OR (physical activity adj4 adherence).ti,ab,kw. OR "motor activit*".ti,ab,kw. OR "physical exercise".ti,ab,kw. OR "home-based exercise".ti,ab,kw. OR "isometric exercise".ti,ab,kw. OR "aerobic exercise".ti,ab,kw. OR "exercise training".ti,ab,kw. OR "exercise program*".ti,ab,kw. OR (exercise adj4 adherence).ti,ab,kw. OR "strength program*".ti,ab,kw. OR "resistance training*".ti,ab,kw. OR "muscle training".ti,ab,kw. OR "strength training".ti,ab,kw.	580,134
**Outcome<br>Sleep Quality<br>#5**	sleep hygiene/ OR sleep/ OR sleep deprivation/ OR sleep quality/ OR "sleep experience".ti,ab,kw. OR "sleep maintenance".ti,ab,kw. OR "sleep quantity".ti,ab,kw. OR "sleeping behavi*".ti,ab,kw. OR "sleep disturbanc*".ti,ab,kw. OR "sleep habit*" .ti,ab,kw. OR "sleep insufficienc*".ti,ab,kw. OR "sleep fragmentation".ti,ab,kw. OR (sleep adj2 trouble).ti,ab,kw.	204,913

	OR (sleeping adj2 troubles).ti,ab,kw. OR "sleep hygiene".ti,ab,kw. OR (sleep adj2 pattern).ti,ab,kw. OR (sleep adj2 patterns).ti,ab,kw. OR "sleep deprivation*".ti,ab,kw. OR (sleep adj2 quality).ti,ab,kw.	
**Other**<br>**#6**	(exp animal/) NOT (exp human/)	5,208,765
**#7**	exp meta analysis/ OR (review OR meta-analysis).ti,ab,kw. OR (review OR meta-analysis OR letter OR editorial OR comment).pt.	6,839,124
**Total**	(#1 AND #2 AND (#3 OR #4 OR #5)) NOT #6<br>((#1 AND #2 AND (#3 OR #4 OR #5)) NOT #6) NOT #7	4,760<br>3,850

**CINAHL**<br>**06/02/2024**		
	**Search query**	**Search results**
**Domain**<br>**#1**	(MH "Osteoarthritis, Hip") OR (MH "Osteoarthritis, Knee") OR ((MH "Hip Joint") OR (MH "Hip") OR (MH "Knee Joint") OR (MH "Knee")) AND (MH "Osteoarthritis") OR ((TI(hip) OR AB(hip) OR TI(hips) OR AB(hips) OR TI(knee) OR AB(knee) OR TI(knees) OR AB(knees) OR TI(lower limb*) OR AB(lower limb*) OR TI(lower extremit*) OR AB(lower extremit*)) AND (TI(osteoarthr*) OR AB(osteoarthr*) OR TI(osteo-arthr*) OR AB(osteo-arthr*) OR TI(arthrosis) OR AB(arthrosis) OR TI(arthroses) OR AB(arthroses) OR TI(degenerative arthr*) OR AB(degenerative arthr*) OR TI(coxarthr*) OR AB(coxarthr*) OR TI(cox-arthr*) OR AB(cox-arthr) OR TI(gonarthr*) OR AB(gonarthr*)))	30,654
**Determinant**<br>**#2**	(MH "Risk Factors") OR TI(risk*) OR AB(risk*) OR TI(factor*) OR AB(factor*) OR TI(facilitat*) OR AB(facilitat*) OR TI(barrier*) OR AB(barrier*) OR TI(determin*) OR AB(determin*) OR TI(predict*) OR AB(predict*)	2,261,562
**Outcome**<br>**Weight**<br>**Management**<br>**#3**	(MH "Diet") OR (MH "Diet, Reducing") OR (MH "Energy Intake") OR TI(eating habit*) OR AB(eating habit*) OR TI(eating behavi*) OR AB(eating behavi*) OR TI(weight N2 management) OR AB(weight N2 management) OR TI(weight N2 maintenance) OR AB(weight N2 maintenance) OR TI(weight N2 reduction) OR AB(weight N2 reduction) OR TI(weight N2 control) OR AB(weight N2 control) OR TI(weight N2 loss) OR AB(weight N2 loss) OR TI(lost weight) OR AB(lost weight) OR ((TI(weight) OR AB(weight)) AND (TI(adherence) OR AB(adherence)))	132,697
**Outcome**<br>**Physical**<br>**Activity**	(MH "Exercise") OR (MH "Therapeutic Exercise") OR (MH "Resistance Training") OR TI(physical activit*) OR AB(physical activit*) OR TI(physical activity N4	169,749

**#4**	adherence) OR AB(physical activity N4 adherence) OR TI(motor activit*) OR AB(motor activit*) OR TI(physical exercise) OR AB(physical exercise) OR TI(home-based exercise) OR AB(home-based exercise) OR TI(isometric exercise) OR AB(isometric exercise) OR TI(aerobic exercise) OR AB(aerobic exercise) OR TI(exercise training) OR AB(exercise training) OR TI(exercise therapy) OR AB(exercise therapy) OR TI(kinesiotherapy) OR AB(kinesiotherapy) OR TI(exercise program*) OR AB(exercise program*) OR TI(exercise N4 adherence) OR AB(exercise N4 adherence) OR TI(strength program*) OR AB(strength program*) OR TI(resistance training*) OR AB(resistance training*) OR TI(muscle training) OR AB(muscle training) OR TI(strength training) OR AB(strength training)	
**Outcome Sleep Quality #5**	(MH "Sleep Hygiene") OR (MH "Sleep") OR (MH "Sleep Deprivation") OR (MH "Sleep Quality") OR TI(sleep experience) OR AB(sleep experience) OR TI(sleep maintenance) OR AB(sleep maintenance) OR TI(sleep quantity) OR AB(sleep quantity) OR TI(sleeping behavi*) OR AB(sleeping behavi*) OR TI(sleep disturbanc*) OR AB(sleep disturbanc*) OR TI(sleep habit*) OR AB(sleep habit*) OR TI(sleep insuffienc*) OR AB(sleep insuffienc*) OR TI(sleep fragmentation) OR AB(sleep fragmentation) OR TI(sleep N2 trouble) OR AB(sleep N2 trouble) OR TI(sleeping N2 troubles) OR AB(sleeping N2 troubles) OR TI(sleep hygiene) OR AB(sleep hygiene) OR TI(sleep N2 pattern) OR AB(sleep N2 pattern) OR TI(sleep N2 patterns) OR AB(sleep N2 patterns) OR TI(sleep deprivation*) OR AB(sleep deprivation*) OR TI(sleep N2 quality) OR AB(sleep N2 quality)	45,680
**Other #6**	(MH "Animals+") NOT (MH "Human+")	93,094
**#7**	TI(review) OR AB(review) OR PT(review) OR (MH "Meta Analysis+") OR TI(meta-analysis) OR AB(meta-analysis) OR PT(meta analysis) OR PT(letter) OR PT(editorial) OR PT(comment)	1,477,865
**Total**	(#1 AND #2 AND (#3 OR #4 OR #5)) NOT #6	1,304
	((#1 AND #2 AND (#3 OR #4 OR #5)) NOT #6) NOT #7	1,070

**PsycINFO 06/02/2024**		
	**Search query**	**Search results**
**Domain #1**	(((DE "Hips") OR (DE "Knee")) AND (DE "Arthritis")) OR ((TI "hip" OR AB "hip" OR TI "hips" OR AB "hips" OR TI "knee" OR AB "knee" OR TI "knees" OR AB "knees" OR TI	1,291

	"lower limb*" OR AB "lower limb*" OR TI "lower extremit*" OR AB "lower extremit*") AND (TI "osteoarthr*" OR AB "osteoarthr*" OR TI "osteo-arthr*" OR AB "osteo-arthr*" OR TI "arthrosis" OR AB "arthrosis" OR TI "arthroses" OR AB "arthroses" OR TI "degenerative arthr*" OR AB "degenerative arthr*" OR TI "coxarthr*" OR AB "coxarthr*" OR TI "cox-arthr*" OR AB "cox-arthr" OR TI "gonarthr*" OR AB "gonarthr*"))	
**Determinant #2**	(DE "Risk Factors") OR TI "risk" OR AB "risk" OR TI "factor" OR AB "factor" OR TI "facilitat*" OR AB "facilitat*" OR TI "barrier" OR AB "barrier" OR TI "determin*" OR AB "determin*" OR TI "predict*" OR AB "predict*"	1,656,527
**Outcome Weight Management #3**	(DE "Diets") OR TI "eating habit*" OR AB "eating habit*" OR TI "eating behavi*" OR AB "eating behavi*" OR TI "weight NEAR/2 management" OR AB "weight NEAR/2 management" OR TI "weight NEAR/2 maintenance" OR AB "weight NEAR/2 maintenance" OR TI "weight NEAR/2 reduction" OR AB "weight NEAR/2 reduction" OR TI "weight NEAR/2 control" OR AB "weight NEAR/2 control" OR TI "weight NEAR/2 loss" OR AB "weight NEAR/2 loss" OR TI "lost weight" OR AB "lost weight" OR ((TI "weight" OR AB "weight") AND (TI "adherence" OR AB "adherence"))	28,320
**Outcome Physical Activity #4**	(DE "Exercise") OR (DE "Exercise Therapy") OR TI "physical activit*" OR AB "physical activit*" OR TI "physical activity NEAR/4 adherence" OR AB "physical activity NEAR/4 adherence" OR TI "motor activit*" OR AB "motor activit*" OR TI "physical exercise" OR AB "physical exercise" OR TI "home-based exercise" OR AB "home-based exercise" OR TI "isometric exercise" OR AB "isometric exercise" OR TI "aerobic exercise" OR AB "aerobic exercise" OR TI "exercise training" OR AB "exercise training" OR TI "exercise therapy" OR AB "exercise therapy" OR TI "kinesiotherapy" OR AB "kinesiotherapy" OR TI "exercise program*" OR AB "exercise program*" OR TI "exercise NEAR/4 adherence" OR AB "exercise NEAR/4 adherence" OR TI "strength program*" OR AB "strength program*" OR TI "resistance training*" OR AB "resistance training*" OR TI "muscle training" OR AB "muscle training" OR TI "strength training" OR AB "strength training"	77,542
**Outcome Sleep Quality #5**	(DE "Sleep") OR (DE "Sleep Deprivation") OR (DE "Sleep Quality") OR TI "sleep experience" OR AB "sleep experience" OR TI "sleep maintenance" OR AB "sleep maintenance" OR TI "sleep quantity" OR AB "sleep quantity" OR TI "sleeping behavi*" OR AB "sleeping	49,481

	behavi*" OR TI "sleep disturbanc*" OR AB "sleep disturbanc*" OR TI "sleep habit*" OR AB "sleep habit*" OR TI "sleep insuffienc*" OR AB "sleep insuffienc*" OR TI "sleep fragmentation" OR AB "sleep fragmentation" OR TI "sleep NEAR/2 trouble" OR AB "sleep NEAR/2 trouble" OR TI "sleeping NEAR/2 troubles" OR AB "sleeping NEAR/2 troubles" OR TI "sleep hygiene" OR AB "sleep hygiene" OR TI "sleep NEAR/2 pattern" OR AB "sleep NEAR/2 pattern" OR TI "sleep NEAR/2 patterns" OR AB "sleep NEAR/2 patterns" OR TI "sleep deprivation*" OR AB "sleep deprivation*" OR TI "sleep NEAR/2 quality" OR AB "sleep NEAR/2 quality"	
**Other**<br>**#6**<br>**#7**	~~(DE "Animals")~~<br><br>TI "review" OR AB "review" OR PT "review" OR (DE "Meta Analysis+") OR TI "meta-analysis" OR AB "meta-analysis" OR PT "meta analysis" OR PT "letter" OR PT "editorial" OR PT "comment"	 <br>445,869
**Total**	~~(#1 AND #2 AND (#3 OR #4 OR #5)) NOT #6~~<br>(#1 AND #2 AND (#3 OR #4 OR #5)) ~~NOT #6~~ NOT #7	 112

**Web of Science 06/02/2024**		
	**Search query**	**Search results**
**Domain**<br>**#1**	TS=(((Hip OR hips OR Knee OR knees OR "lower limb*" OR "lower extremit*") AND (Osteoarthr* OR "Osteo-arthr*" OR Arthrosis OR Arthroses OR "Degenerative arthr*" OR Coxarthr* OR "Cox-arthr*" OR Gonarthr*)))	76,546
**Determinant**<br>**#2**	TS=(Risk* OR Factor* OR Facilitat* OR Barrier* OR Determin* OR Predict*)	18,913,356
**Outcome Weight Management**<br>**#3**	TS=("Eating habit*" OR "Eating behavi*" OR (Weight NEAR/2 management) OR (Weight NEAR/2 maintenance) OR (Weight NEAR/2 reduction) OR (Weight NEAR/2 control) OR (Weight NEAR/2 loss) OR "Lost weight" OR ("Weight" AND "Adherence"))	262,817
**Outcome Physical Activity**<br>**#4**	TS=("Physical activit*" OR (Physical activity NEAR/4 adherence) OR "Motor activit*" OR "Physical exercise" OR "Home-based exercise" OR "Isometric exercise" OR "Aerobic exercise" OR "Exercise training" OR "Exercise therapy" OR "Kinesiotherapy" OR "Exercise program*" OR (Exercise NEAR/4 adherence) OR "Strength program*" OR "Resistance training*" OR "Muscle training" OR "Strength training")	346,294

**Outcome Sleep Quality #5**	TS=("Sleep experience*" OR "Sleep maintenance" OR "Sleep quantity" OR "Sleeping behavi*" OR "Sleep disturbanc*" OR "Sleep habit*" OR "Sleep insufficienc*" OR "Sleep fragmentation" OR (Sleep NEAR/2 trouble) OR (Sleeping NEAR/2 troubles) OR "Sleep hygiene" OR (Sleep NEAR/2 pattern) OR (Sleep NEAR/2 patterns) OR "Sleep deprivation*" OR (Sleep NEAR/2 quality))	79,122
**Other #6**	~~Animal NOT Human~~	-
**#7**	DT=(Review OR Letter OR Editorial Material) OR TS=(Review OR Meta-analysis)	8,966,002
**Total**	~~(#1 AND #2 AND (#3 OR #4 OR #5)) NOT #6~~	-
	(#1 AND #2 AND (#3 OR #4 OR #5)) ~~NOT#6~~ NOT #7	2,693
