# Supplementary material for: Psychosocial factors associated with physical activity, weight management, and sleep in adults with hip and knee osteoarthritis: a systematic review
Source: BMC Rheumatol. 2025 May 9;9:51. doi: 10.1186/s41927-025-00506-x (PMC12063410; doi:10.1186/s41927-025-00506-x)
Supplement: Supplementary file 2 — Supplementary Material 2: Additional file 2 - Methodological quality.pdf. Assessment of methodological quality of included studies using the NHLBI tool by researcher BD [file 41927_2025_506_MOESM2_ESM.pdf]

Additional file 2. Methodological quality assessment of included studies

First author (publication year)	1	2	3	4	5	6[1]	7[1]	8	9[1]	10	11[1]	12	13	14	Rating[2]
Acar (2022)	YES	YES	CD	YES	NO	NO	NO	NA	YES	NO	YES	YES	NA	NO	FAIR
Akintayo (2019)	YES	YES	CD	YES	YES	NO	NO	NA	YES	NO	YES	CD	NA	NO	FAIR
Aydemir (2022)	YES	YES	CD	YES	NO	NO	NO	YES	YES	NO	YES	YES	NA	YES	FAIR
Aydemir (2023)	YES	YES	CD	YES	NO	NO	NO	YES	YES	NO	YES	YES	NA	YES	FAIR
Choi (2014)	YES	YES	YES	YES	NO	YES	NO	NA	NO	YES	YES	YES	YES	YES	FAIR
Degerstedt (2020)	YES	YES	CD	YES	NO	YES	YES	YES	YES	NO	NO	NO	YES	YES	FAIR
Di Maio (2020)	YES	YES	CD	YES	YES	YES	NO	YES	YES	NO	YES	NO	NA	YES	FAIR
Duarte (2022)	YES	YES	CD	YES	NO	NO	NO	YES	YES	NO	YES	YES	NA	NO	FAIR
Fawzy (2022)	YES	YES	NO	YES	NO	NO	NO	NA	YES	NO	YES	YES	NA	NO	POOR
Goff (2024)	YES	YES	CD	YES	YES	NO	NO	NO	YES	NO	YES	YES	NA	YES	FAIR
Hamdi (2021)	YES	YES	CD	YES	NO	NO	NO	YES	YES	NO	YES	YES	NA	YES	FAIR
Hanrungcharatorn (2017)	YES	YES	CD	YES	YES	NO	NO	YES	YES	NO	YES	CD	NA	NO	FAIR
Hawker (2010)	YES	YES	CD	YES	NO	NO	NO	NO	YES	NO	NO	YES	NO	YES	POOR
Heesch (2011)	YES	YES	NO	NO	NO	NO	NO	YES	YES	NO	YES	YES	NA	YES	FAIR
Hsu (2022)	YES	YES	CD	YES	NO	NO	NO	YES	YES	NO	YES	YES	NA	YES	FAIR
Kilinc (2019)	YES	YES	CD	YES	NO	NO	NO	YES	NO	NO	YES	CD	NA	NO	POOR
Mahgoub (2020)	YES	YES	CD	YES	NO	NO	NO	YES	YES	NO	YES	CD	NA	NO	FAIR
Martire (2013)	YES	YES	NO	NO	NO	NO	NO	YES	YES	NO	NO	YES	YES	YES	POOR
Murphy (2013)	YES	YES	YES	YES	NO	YES	NO	YES	YES	NO	YES	CD	YES	YES	GOOD

Nemati (2023)	YES	YES	CD	YES	NO	NO	NO	YES	YES	YES	YES	CD	NR	YES	FAIR
O’Brien (2016)	YES	YES	CD	YES	NO	YES	YES	YES	NO	YES	YES	CD	NA	YES	POOR
Odole (2022)	YES	NO	CD	CD	NO	NO	NO	YES	YES	NO	YES	YES	NA	NO	FAIR
Parmelee (2015)	YES	YES	CD	NO	NO	YES	YES	YES	YES	YES	YES	CD	NO	YES	GOOD
Rosemann (2007)	YES	YES	YES	YES	NO	NO	NO	YES	YES	NO	YES	CD	NA	YES	FAIR
Taylor (2018)	YES	YES	CD	YES	NO	NO	NO	YES	YES	NO	YES	YES	NA	YES	FAIR
Uritani (2020)	YES	YES	NO	YES	NO	YES	NO	YES	YES	NO	YES	CD	NA	YES	FAIR
White (2012)	YES	YES	CD	YES	NO	NO	NO	NO	YES	NO	YES	CD	NA	YES	POOR
Wolf (2010)	YES	NO	CD	NR	NO	YES	YES	YES	NO	NO	YES	CD	NO	NO	FAIR
Zhaoyang (2017)	YES	YES	NO	NO	NO	YES	NO	YES	NO	YES	YES	CD	YES	YES	POOR
Zhaoyang (2019)	YES	YES	CD	YES	NO	YES	NO	YES	NO	YES	YES	CD	YES	YES	FAIR
Zhaoyang (2020)	YES	YES	CD	YES	NO	YES	NO	YES	YES	YES	YES	CD	YES	YES	GOOD

[1]These criteria had the focus of the methodological quality assessment.

[2]The final ratings were determined in accordance by both researchers (BD, BC) after a discussion. The ratings on the single items (1 – 14) shown in this table were from the first researcher (BD).

CD = cannot determine; NA = not applicable; NR = not reported
