# Supplementary material for: Psychosocial factors associated with physical activity, weight management, and sleep in adults with hip and knee osteoarthritis: a systematic review
Source: BMC Rheumatol. 2025 May 9;9:51. doi: 10.1186/s41927-025-00506-x (PMC12063410; doi:10.1186/s41927-025-00506-x)
Supplement: Supplementary file 3 — Supplementary Material 3: Additional file 3 - Psychosocial determinants.pdf. Extracted data of study results concerning the relationship between a psychosocial determinant and one of the outcome behaviors (physical activity, weight management or sleep) [file 41927_2025_506_MOESM3_ESM.pdf]

Additional file 3. Psychosocial determinants of physical activity, weight management, and sleep

Psychosocial determinants of physical activity

**Determinant**	**First author (publication year)**	**N**	**Outcome**	**Timepoint/Follow-up period**	**Effect size**	**Strength of association[1]**	**Bivariate or multivariate analyses[2]**
Barriers to physical activity	Heesch (2011)	485	Meeting Arthritis-Specific Leisure-Time Physical Activity Recommendations[3]	One timepoint	OR = .78 [0.56-1.09], *p* = .147	None	Multivariate
			Strengthening exercises	One timepoint	OR = .69 [0.49-0.97], *p* = .035	Weak (-)	Multivariate
Benefits of physical activity	Heesch (2011)	485	Stretching exercises	One timepoint	OR = 1.62 [1.09-2.40], *p* = .016	Weak (+)	Multivariate
			Strengthening exercises	One timepoint	OR = 1.29 [0.88-1.82], *p* = .192	None	Multivariate
Depressive symptoms	Di Maio (2020)	238	Daily MVPA[4]	One timepoint	*r* = -.05	None	Bivariate
			Daily steps	One timepoint	*r* = -.08	None	Bivariate
	Hsu (2022)	188	PASE scores[5]: comparison	One timepoint	*p* = .06	None	Bivariate

			between active and inactive groups				
	Klininc (2019)	200	MET-minute/week[6]	One timepoint	*r* = -.43, *p* = .00	Moderate (-)	Bivariate
	Murphy (2013)	172	Activity counts per minute	One timepoint	*r* = -.03	None	Bivariate
	Nemati (2023)	2603	PASE scores[5]	Baseline	*r* = -.01	None	Bivariate
		1498	PASE scores[5]	Follow-up after 8 years	*r* = -.08, *p* < *.05*	Weak (-)	Bivariate
	Rosemann (2007)	427	MET-minute[6]	One timepoint	*r* = .14, *p* < .001	Weak (+)	Bivariate
		594	MET-minute[6]	One timepoint	*r* = .16, *p* = .020	Weak (+)	Bivariate
	Uritani (2020)	167	Steps per day	One timepoint	β = -59 [-138, 19], *p* = .138, Adjusted R^2 = .34	None	Multivariate
	White (2012)	99	Steps per day	One timepoint	β = -59.5 [-1137.7; 1018.7] Adjusted β = 85.2 [-871.6; 1042.1]	(-)	Multivariate
Intention	Duarte (2022)	41	MVPA[4]	One timepoint	β = 119.21 [43.764, 194.661], *p* = .003	(+)	Bivariate

	O’Brien (2016)	1	Step count	Baseline	$r = -.69$, $p < .001$	Strong (-)	Bivariate
				One day later	$r = .75$, $p < .001$	Strong (+)	Bivariate
				Two days later	$r = -.65$, $p < .001$	Strong (-)	Bivariate
Kinesiophobia	Acar (2022)	60	MET-minute/week[6]	One timepoint	$r = -.05$, $p = .68$	None	Bivariate
	Aydemir (2022)	37	UCLA score[7]	One timepoint	$r = -.77$, $p < .01$	Very strong (-)	Bivariate
	Aydemir (2023)	40	UCLA score[7]	One timepoint	$r = -.70$, $p < .001$	Very strong (-)	Bivariate
	Goff (2024)	425	UCLA score[7]	One timepoint	$r = -.22$, $p < .001$	Weak (-)	Bivariate
	Kilinc (2019)	200	MET-minute/week[6]	One timepoint	$r = -.69$, $p = .00$	Strong (-)	Bivariate
	Uritani (2020)	167	Steps per day	One timepoint	$\beta = -117$ [-227; -8], $p = .036$	(-)	Multivariate
Negative affect	Zhaoyang (2017)	135	Daily steps	One timepoint	$r = -.11$	None	Bivariate
			Daily MVPA[4]	One timepoint	$r = -.05$	None	Bivariate
	Zhaoyang (2019)	143	Daily MVPA[4]	One timepoint	$r = -.04$	None	Bivariate

	Zhaoyang (2020)	143	Daily steps	One timepoint	*r* = -.13	None	Bivariate
			Daily MVPA[4]	One timepoint	*r* = -.07	None	Bivariate
Pain catastrophizing	Hanrungcharatorn (2017)	242	Low and moderate to high PA	One timepoint	β = 0.01, SE = 0.20, OR = 1.01, [0.98; 1.04], *p* > .05	None	Bivariate
	Uritani (2020)	167	Steps per day	One timepoint	β = -44 [-86; -1], *p* = .044	(-)	Multivariate
	Zhaoyang (2020)	143	Daily MVPA[4]	One timepoint	*r* = -.18, *p* < .05	Weak (-)	Bivariate
Pain-related fear	Hanrungcharatorn (2017)	242	Low and moderate to high PA	One timepoint	β = -0.07, SE = 0.02, OR = 0.94, [0.90; 0.98], *p* < .01	Weak (-)	Bivariate
Perceived controllability	O'Brien (2016)	1	Step count	Baseline	*r* = -.55, *p* < .001	Strong (-)	Bivariate
				One day later	*r* = -.38, *p* < .001	Moderate (-)	Bivariate
				Two days later	*r* = .29, *p* < .01	Weak (+)	Bivariate
Positive affect	Zhaoyang (2017)	135	Daily steps	One timepoint	*r* = .09	None	Bivariate
			Daily MVPA[4]	One timepoint	*r* = .14, *p* < .10	None	Bivariate
	Zhaoyang (2019)	143	Daily MVPA[4]	One timepoint	*r* = .13	None	Bivariate

	White (2012)	640	Steps per day	One timepoint	β = 1157.7 [431.2; 1884.1]<br>Adjusted β = 711.0 [55.1; 1366.9]	None	Multivariate
Self-efficacy	Degerstedt (2020)	3135	Days per week of being physically active for ≥30 min	One timepoint	LSM ± SE<br>Low 4.90 ± 0.08<br>High 5.05 ± 0.08<br>$p < .01$	(+)	Multivariate
	Goff (2024)	425	UCLA score[7]	One timepoint	$r = .20$, $p = .004$	Weak (+)	Bivariate
	Heesch (2011)	485	Meeting Arthritis-Specific Leisure-Time Physical Activity Recommendations[3]	One timepoint	OR = 1.15 [0.85-1.55], $p = .369$	None	Multivariate
			Strengthening exercises	One timepoint	OR = 1.26 [0.90-1.76], $p = .185$	None	Multivariate
			Stretching exercises	One timepoint	OR = 1.33 [0.97-1.83], $p = .185$	None	Multivariate
	O'Brien (2016)	1	Step count	Baseline	$r = .27$, $p < .05$	Weak (+)	Bivariate
				One day later	$r = .69$, $p < .001$	Strong (+)	Bivariate
				Two days later	$r = -.67$, $p < .001$	Strong (-)	Bivariate

	Uritani (2020)	167	Steps per day	One timepoint	β = 117 [-12; 246], *p* = .075	None	Multivariate
	Zhaoyang (2017)	135	Daily steps	One timepoint	$r = .25, p < .01$	Weak (+)	Bivariate
			Daily MVPA[4]	One timepoint	$r = .15, p < .10$	None	Bivariate
Social support	Hanrungcharatorn (2017)	242	Low and moderate to high PA	One timepoint	β = 0.09, SE = 0.12, OR = 1.09, [0.87; 1.04], $p > .05$	None	Bivariate
	Zhaoyang (2017)	135	Daily steps	One timepoint	$r = .03$	None	Bivariate
			Daily MVPA[4]	One timepoint	$r = .04$	None	Bivariate
Willpower beliefs	Di Maio (2020)	238	Daily MVPA[4]	One timepoint	$b = 5.55$, SE = 2.43, $p = .023$	(+)	Multivariate
			Daily steps	One timepoint	$b = 559.90$, SE = 245.62, $p = .021$	(+)	Multivariate

[1]Determined by Rosenthal (1996). Qualitative interpretation of the strength of association for Pearson Correlation Coefficient: $r \approx .10$ (weak), $r \approx .30$ (moderate) $r \approx .50$ (strong), $r \approx .70$ (very strong). Qualitative interpretation of the strength of association for Odds Ratio: OR ≈ 1.5 to 1 (weak), OR ≈ 2.5 to 1 (moderate), OR ≈ 4 to 1 (strong), OR ≈ 10 to 1 (very strong). The direction of association is indicated with + (positive) or - (negative). When *r* or OR are not mentioned, only the direction of the significant association is noted with (+) or (-).

[2]Multivariate refers to analyses that involve more than two variables, contrasting with bivariate analyses that consider only two variables (Denis, 2020).

[3]Meeting Arthritis-Specific Leisure-Time Physical Activity Recommendations (LTPA) consist of ≥360 METs[5] per week (computed as ≥30 min x 4 METs x 3 times per week) (Heesch, 2011).

[4]Moderate to vigorous physical activity (MVPA).

[5]Physical Activity Scale for the Elderly (PASE).

[6]Metabolic Equivalent of Task (MET), indicating how much energy an activity consumes compared to resting.

[7]University of California at Los Angeles (UCLA) activity score.

Psychosocial determinants of weight management

Determinant	First author (publication year)	N	Outcome	Timepoint/Follow-up period	Effect size	Strength of association[1]	Bivariate or multivariate analyses[2]
Depressive symptoms	Di Maio (2020)	238	BMI	One timepoint	r = .18, p < .01	Weak (+)	Bivariate
	Mahgoub (2020)	59	BMI	One timepoint	r = .36, p = .005	Moderate (+)	Bivariate
		32	BMI	One timepoint	r = .29, p = .11	None	Bivariate
	Murphy (2013)	172	BMI	One timepoint	r = .01	None	Bivariate
	Wolf (2010)	89	Weight loss in pounds	Follow-up (16 weeks)	r = -.41, p < .001	Moderate (-)	Bivariate
		75	Weight loss in pounds	Follow-up (32 weeks)	r = -.31, p = .01	Moderate (-)	Bivariate
Kinesiophobia	Aydemir (2022)	37	BMI	One timepoint	r = .13	None	Bivariate
	Odole (2022)	77	BMI	One timepoint	r = .06, p = .60	None	Bivariate
Motivation	Wolf (2010)	84	Weight loss in pounds	Follow-up (16 weeks)	r = .12, p = .29	None	Bivariate
		70	Weight loss in pounds	Follow-up (32 weeks)	r = .23, p = .06	None	Bivariate

Negative mood	Choi (2014)	54	Calorie intake	Two-day diary	$p = .01$	(-)	Multivariate
		54	Fat intake	Two-day diary	$p = .03$	(-)	Multivariate
		54	Sugar intake	Two-day diary	$p = .01$	(-)	Multivariate
Pain anxiety	Mahgoub (2020)	59	BMI	One timepoint	$r = .03$, $p = .84$	None	Bivariate
		32	BMI	One timepoint	$r = .04$, $p = .84$	None	Bivariate
Pain catastrophizing	Odole (2022)	77	BMI	One timepoint	$r = .35$, $p = .00$	Moderate (+)	Bivariate
Self-efficacy	Odole (2022)	77	BMI	One timepoint	$r = -.30$, $p = .01$	Moderate (-)	Bivariate
	Wolf (2010)	85	Weight loss in pounds	Follow-up (16 weeks)	$r = .03$, $p = .81$	None	Bivariate
		79	Weight loss in pounds	Follow-up (32 weeks)	$r = .07$, $p = .57$	None	Bivariate
Willpower beliefs	Di Maio (2020)	238	BMI	One timepoint	$r = -.15$, $p < .01$	Weak (-)	Bivariate

[1]Determined by Rosenthal (1996). Qualitative interpretation of the strength of association for Pearson Correlation Coefficient: $r \approx .10$ (weak), $r \approx .30$ (moderate) $r \approx .50$ (strong), $r \approx .70$ (very strong). Qualitative interpretation of the strength of association for Odds Ratio: OR ≈ 1.5 to 1 (weak), OR ≈ 2.5 to 1 (moderate), OR ≈ 4 to 1 (strong), OR ≈ 10 to 1 (very strong). The direction of association is indicated with + (positive) or - (negative). When *r* or OR are not mentioned, only the direction of the significant association is noted with (+) or (-).

[2]Multivariate refers to analyses that involve more than two variables, contrasting with bivariate analyses that consider only two variables (Denis, 2020).

Psychosocial determinants of sleep

Determinant	First author (publication year)	N	Outcome	Timepoint/Follow-up period	Effect size	Strength of association[1]	Bivariate or multivariate analyses[2]
Anxiety	Hamdi (2021)	40	Sleep problems	One timepoint	$r = .60$, $p = .003$	Strong (+)	Bivariate
Couple closeness	Martire (2013)	138	Sleep quality	22-day diary	$r = .01$, $p < .05$	Weak (+)	Bivariate
Depressive symptoms	Akintayo (2019)	250	Poor sleep quality[3]	One timepoint	β = .238 [1.131; 1.423], OR = 1.269, $p < .001$	Weak (+)	Multivariate
	Fawzy (2022)	20	Insomnia	One timepoint	$r = .51$, $p = .02$	Strong (+)	Bivariate
	Hamdi (2021)	40	Sleep problems	One timepoint	$r = .30$, $p = .059$	Moderate (+)	Bivariate
	Hawker (2010)	577	Poor sleep quality[3]	One timepoint	Adjusted OR = 1.09 [1.05; 1.13], $p < .0001$	Weak (+)	Multivariate
	Mahgoub (2020)	59	Poor sleep quality[3]	One timepoint	$r = .33$, $p = .01$	Moderate (+)	Bivariate
		32	Poor sleep quality[3]	One timepoint	$r = .18$, $p = .32$	None	Bivariate
	Murphy (2013)	172	Sleep efficiency	One timepoint	$r = -.28$, $p < .05$	Weak (-)	Bivariate

	Parmelee (2015)	367	Sleep disturbance	Baseline	*r* = .27, β = .206 [.110; .302], *p* < .001	Weak (+)	Multivariate
		288	Sleep disturbance	Follow-up (1 year)	*r* = .32, β = .158 [.072; .243], *p* < .001	Moderate (+)	Multivariate
	Taylor (2018)	299	Insomnia	One timepoint	Est (SE) = 1.10 (0.06) [0.99; 1.22], *p* = .001	(+)	Bivariate
Pain anxiety	Mahgoub (2020)	59	Poor sleep quality[3]	One timepoint	*r* = .38, *p* = .003	Moderate (+)	Bivariate
		32	Poor sleep quality[3]	One timepoint	*r* = .40, *p* = .02	Moderate (+)	Bivariate
PTSD	Taylor (2018)	299	Insomnia	One timepoint	Est (SE) = 5.56 (0.98) [3.73; 7.57], *p* < .001	(+)	Bivariate

[1]Determined by Rosenthal (1996). Qualitative interpretation of the strength of association for Pearson Correlation Coefficient: *r* ≈ .10 (weak), *r* ≈ .30 (moderate) *r* ≈ .50 (strong), *r* ≈ .70 (very strong). Qualitative interpretation of the strength of association for Odds Ratio: OR ≈ 1.5 to 1 (weak), OR ≈ 2.5 to 1 (moderate), OR ≈ 4 to 1 (strong), OR ≈ 10 to 1 (very strong). The direction of association is indicated with + (positive) or - (negative). When *r* or OR are not mentioned, only the direction of the significant association is noted with (+) or (-).

[2]Multivariate refers to analyses that involve more than two variables, contrasting with bivariate analyses that consider only two variables (Denis, 2020).

[3]PSQI ≥ 5

References

Denis, D. J. (2020). Univariate, bivariate, and multivariate statistics using R: quantitative tools for data analysis and data science. John Wiley & Sons.

Rosenthal, J. A. (1996). Qualitative descriptors of strength of association and effect size. Journal of social service Research, 21(4), 37-59
